# Stimulant Reduction Intervention using Dosed Exercise (STRIDE) - CTN 0037: Study protocol for a randomized controlled trial

**DOI:** 10.1186/1745-6215-12-206

**Published:** 2011-09-19

**Authors:** Madhukar H Trivedi, Tracy L Greer, Bruce D Grannemann, Timothy S Church, Eugene Somoza, Steven N Blair, Jose Szapocznik, Mark Stoutenberg, Chad Rethorst, Diane Warden, Kolette M Ring, Robrina Walker, David W Morris, Andrzej S Kosinski, Tiffany Kyle, Bess Marcus, Becca Crowell, Neal Oden, Edward Nunes

**Affiliations:** 1 The University of Texas Southwestern Medical Center at Dallas, 5323 Harry Hines Blvd., Dallas, Texas, 75390-9119, USA; 2 Preventive Medicine Laboratory, Pennington Biomedical Research Center, 6400 Perkins Rd, Baton Rouge, LA, 70808, USA; 3 Cincinnati Addictions Research Center (CinARC); Department of Psychiatry, University of Cincinnati College of Medicine, 260 Stetson St., Cincinnati, OH, 45219, USA; 4 Department of Exercise Science, University of South Carolina, 514 Main Street, Columbia, SC, 29208 USA; 5 Department of Epidemiology and Biostatistics, University of South Carolina, 800 Sumter Street, Columbia, SC, 29208, USA; 6 Department of Epidemiology and Public Health, University of Miami, Miller School of Medicine, 1120 NW 14th Street, Miami, 33136 FL, USA; 7 Department of Biostatistics and Bioinformatics, Duke University Medical Center, Duke Clinical Research Institute, P. O. Box 17969, Durham, NC 27715, USA; 8 The Center for Drug-Free Living, 3670 Maguire Boulevard, Orlando, FL, 32853, USA; 9 Department of Family and Preventive Medicine, The University of California, San Diego, 9500 Gilman Drive, 0622, La Jolla, California., 92093-0622, USA; 10 Nexus Recovery Center, Inc., 8733 La Prada Dr. Dallas, Texas, 75228, USA; 11 The EMMES Corporation, 401 N. Washington St., Rockville, MD, 20850, USA; 12 New York State Psychiatric Institute/Department of Psychiatry, College of Physicians and Surgeons of Columbia University, 1051 Riverside Drive, Unit 69, New York, NY 10032, USA

**Keywords:** stimulant abuse, stimulant dependence, exercise, health education, behavioral intervention

## Abstract

**Background:**

There is a need for novel approaches to the treatment of stimulant abuse and dependence. Clinical data examining the use of exercise as a treatment for the abuse of nicotine, alcohol, and other substances suggest that exercise may be a beneficial treatment for stimulant abuse, with direct effects on decreased use and craving. In addition, exercise has the potential to improve other health domains that may be adversely affected by stimulant use or its treatment, such as sleep disturbance, cognitive function, mood, weight gain, quality of life, and anhedonia, since it has been shown to improve many of these domains in a number of other clinical disorders. Furthermore, neurobiological evidence provides plausible mechanisms by which exercise could positively affect treatment outcomes. The current manuscript presents the rationale, design considerations, and study design of the National Institute on Drug Abuse (NIDA) Clinical Trials Network (CTN) CTN-0037 Stimulant Reduction Intervention using Dosed Exercise (STRIDE) study.

**Methods/Design:**

STRIDE is a multisite randomized clinical trial that compares exercise to health education as potential treatments for stimulant abuse or dependence. This study will evaluate individuals diagnosed with stimulant abuse or dependence who are receiving treatment in a residential setting. Three hundred and thirty eligible and interested participants who provide informed consent will be randomized to one of two treatment arms: Vigorous Intensity High Dose Exercise Augmentation (DEI) or Health Education Intervention Augmentation (HEI). Both groups will receive TAU (i.e., usual care). The treatment arms are structured such that the quantity of visits is similar to allow for equivalent contact between groups. In both arms, participants will begin with supervised sessions 3 times per week during the 12-week acute phase of the study. Supervised sessions will be conducted as one-on-one (i.e., individual) sessions, although other participants may be exercising at the same time. Following the 12-week acute phase, participants will begin a 6-month continuation phase during which time they will attend one weekly supervised DEI or HEI session.

**Clinical Trials Registry:**

ClinicalTrials.gov, NCT01141608

http://clinicaltrials.gov/ct2/show/NCT01141608?term=Stimulant+Reduction+Intervention+using+Dosed+Exercise&rank=1

## Background

There is a need for novel approaches to the treatment of stimulant use disorders. Stimulant use disorders are chronic, relapsing illnesses with few highly efficacious treatments [1]. In control conditions (Treatment as Usual; TAU) for substance use disorders, typically only about 13% of participants achieve abstinence [1]. Abstinence rates for treatments designed to augment TAU vary widely - ranging from 14-60%[2-5] - depending on the outcome variable and primary endpoint selected. Currently, the best treatments for cocaine and other stimulant use disorders are behavioral treatments that combine cognitive behavioral therapy (CBT) with contingency management [1,6]. However, it is clear that new treatments are still needed for stimulant abuse and dependence.

Exercise is a promising new treatment option for stimulant abuse and dependence. Randomized controlled trials examining exercise to improve outcomes in smoking cessation provide some of the most convincing support for investigating the use of exercise (and most frequently, vigorous intensity exercise) to improve outcomes in stimulant abuse [7-11]. While randomized controlled trials in patients abusing substances other than tobacco or alcohol are not yet available, some studies report benefits such as increased abstinence and reduced substance use that are associated with the use of exercise [12-14]. Furthermore, in a post hoc analysis of data from 187 participants in two randomized trials evaluating contingency management for the treatment of substance abuse disorders [15,16], participants who reported engaging in exercise-related activities had an increased length of abstinence [17]. In a recent pilot study of moderate-intensity aerobic exercise added to treatment for 16 individuals with substance dependence, percent days abstinent significantly increased from baseline, and 66.7% of the sample had been continuously abstinent at the end of a 12-week intervention [18].

Exercise may also provide additional health benefits and functional improvements for stimulant users. Exercise has been shown to reduce depression and anxiety during alcohol treatment [19-21], as well as smoking cessation treatment [22-24]. Exercise is also associated with improved quality of life [25] and sleep [26], both of which are disrupted by stimulant use even after abstinence is achieved [27,28]. In addition, concern about weight gain following cessation of abused substances may increase risk of substance use relapse [29-31], and regular exercise may prevent or reduce post cessation weight gain. Furthermore, Greenwood et al. [32] has demonstrated improvements in hippocampal-dependent contextual learning and memory in rats with exercise. Similar results have been found for exercise-induced hippocampal neurogenesis and improvements in spatial memory in rats and mice [33-35]. Since substance abuse has been associated with memory impairments that are influenced by hippocampal function [36], exercise may be associated with improved memory for these individuals.

Exercise may improve outcomes through any of several possible mechanisms. Exercise is likely to impact the underlying biology of addicted persons, as well as act as a behavioral treatment intervention. Summarizing studies of the effect of exercise on neurotransmitters [37] conclude that exercise results in changes in synthesis and metabolism within central dopaminergic, noradrenergic, and serotonergic systems, all of which are implicated in addiction. Additional biological effects of exercise may include decreased reactivity to stress [38] and decreased use of substances as a way of coping with stress [39]. Exercise-induced improvements in self-efficacy [40,41] may be another mechanism for improving outcomes. It has also been suggested that exercise may be a distraction [42], allowing attention to be diverted from urges to use substances [43] or a positive lifestyle change that can substitute for use of substances [44,45].

The current paper provides a description of the design of the Stimulant Reduction Intervention using Dosed Exercise (STRIDE) study, a multisite randomized, controlled trial aimed at comparing the augmentation of treatment as usual with either an exercise or health education intervention in a stimulant abusing population. If exercise is found to improve outcomes for substance use disorders, the public health significance would be substantial. A novel, low cost treatment would be available for substance users that could assist with both acute treatment and reducing long-term relapse.

There were several challenges in the design of such a trial, such as: 1) determining the appropriate comparison group, 2) selecting the appropriate primary outcome, and 3) selecting the appropriate setting for the conduct of the study. These challenges were enhanced by the fact that there are no standardized designs that have focused on how best to assess the effects of augmentation treatments, particularly those that take into account patients' transition from residential to intensive outpatient and longer term treatment. This problem is compounded by the fact that there is no agreed upon single outcome to best assess the effectiveness of a treatment for stimulant abuse [46]. This manuscript provides a general description of the STRIDE trial and how the particular issues associated with this type of study were addressed.

## Methods/Design

### Overview

**Figure 1:**
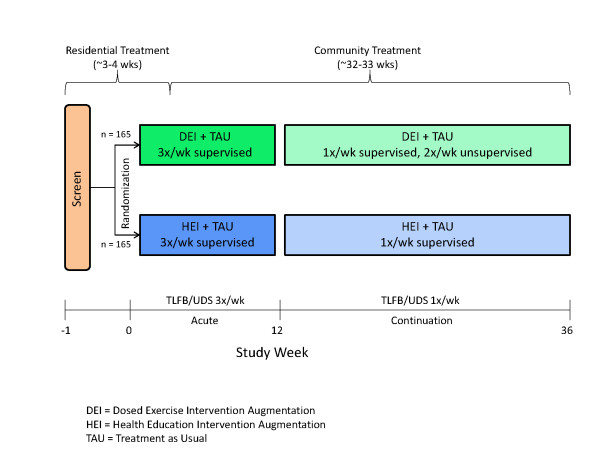
**STRIDE Design Schematic**.

STRIDE is designed to test the efficacy of exercise or health education augmentation for the treatment of stimulant abuse. This study will evaluate individuals diagnosed with stimulant abuse or dependence (cocaine, methamphetamine, amphetamine or other stimulant, except caffeine or nicotine, as defined by the Diagnostic and Statistical Manual of Mental Disorders, Fourth Edition, Text Revision [DSM-IV-TR]) who are receiving treatment in a residential setting. Three hundred and thirty eligible and interested participants who provide informed consent will be randomized to one of two treatment arms: Vigorous Intensity High Dose Exercise Augmentation (DEI) or Health Education Intervention Augmentation (HEI). Both groups will receive TAU (i.e., usual care). The study intervention begins while participants are receiving treatment in a program with a residential stay. The treatment arms are structured such that the quantity of visits is similar to allow for equivalent contact between groups. In both arms, participants will begin with supervised sessions 3 times per week during the 12-week acute phase of the study. Supervised sessions will be conducted as one-on-one (i.e., individual) sessions, although other participants may be exercising at the same time. Following the 12-week acute phase, participants will begin a 6-month continuation phase during which time they will attend one weekly supervised DEI or HEI session. The study design is displayed in Figure 1. A Data and Safety Monitoring Board assembled by NIDA approved the final study design and will be providing ongoing monitoring throughout implementation of the trial.

### Study Sites

This study is being conducted on the National Drug Abuse Treatment Clinical Trials Network (CTN). Participating sites are CTN Community Treatment Programs (CTPs) meeting site characteristics identified based on the requirements of the study protocol [47]. Each site is partnered with a Regional Research and Training Center (RRTC), an academic institution led by a substance use researcher. The RRTC helps guide the conduct of the study at the site. The site characteristics include: 1) a treatment program with a residential component and length of stay generally between 21 and 30 days; 2) no formal exercise program that includes more than an hour a week of exercise, or willingness to exclude participants from the program; 3) availability of community outpatient treatment at or near the residential setting where the study is located so that it is feasible for participants to return to the study site to complete study activities for the full 9 months of the study; 4) a sufficient number of clients admitted to residential treatment per month who might be eligible to participate in the study; and 5) adequate space to accommodate study staff and activities. Sites were selected in two groups based on survey responses, phone interviews, and site visits. Four Wave 1 sites were identified and approved to begin the study about 6 months earlier than the five Wave 2 sites. This model allows for early identification and resolution of any operational issues in the conduct of the study.

### Aims

The primary aim of STRIDE is to compare percent days of abstinence between the DEI and HEI groups based on stimulant use during the 12-week acute phase. It is hypothesized that DEI will be associated with significantly greater (*p *< 0.05) percent days of abstinence than HEI as measured by a corrected Timeline Followback. Timeline Followback (TLFB)[48,49] is collected as described by Sobell and with the aid of a Substance Use Diary. Additionally, a urine drug screen is collected three times per week to improve the accuracy of and correct the self-report data from the TLFB at the time of data analysis based on an algorithm [50].

Secondary aims of STRIDE are: 1) to compare time to relapse (defined as second positive urine test [for stimulants] and use of drugs (assessed via TLFB) between the DEI and HEI groups; 2) to evaluate withdrawal symptoms between the DEI and HEI groups; 3) to evaluate drug use and related outcomes for other substances (categorized as alcohol, cannabinoids, nicotine, opioids, or sedative/hypnotic/anxiolytics); 4) to compare time to dropout from substance abuse treatment between the DEI and HEI groups; 5) to evaluate drug use and related outcomes during the entire course of the study (i.e., randomization to 9 months). Finally, as an exploratory aim, we will attempt to determine if there are additional health benefits to using exercise augmentation in the treatment of substance use disorders. Specifically, we will examine the effects of exercise augmentation on sleep, cognitive function, mood, quality of life, anhedonia, and weight gain compared to HEI over the course of the 12-week acute phase and over the 9-month study period.

### Eligibility Criteria

Participants will be ambulatory males or females, ages 18 to 65, who have been admitted to substance use treatment in a participating residential setting. Participants must have self-reported stimulant drug use (cocaine, methamphetamine, amphetamine, or other stimulant, excluding caffeine and nicotine) within the 30 days prior to admission for residential treatment and meet DSM-IV-TR criteria for substance abuse or dependence for stimulants within the last 12 months. They must receive medical clearance through a protocol-defined maximal exercise test (in accordance with American College of Sports Medicine (ACSM) guidelines [51]) and a careful history and physical examination from protocol-approved medical personnel. Participants must have the ability to understand and willingness to provide written informed consent. Persons meeting the following criteria will be excluded from participation: general medical condition(s) that contraindicate exercise, opiate dependence, psychosis, or other psychiatric issue(s) that poses a safety risk, pregnancy, or concomitant treatment with beta blockers or opioid replacement therapy (e.g., methadone or bupenorphine).

### Exercise Intervention

#### Intervention Overview

Participants in DEI will perform their exercise sessions on a Life Fitness 95T-seriesTreadmill (Schiller Park, IL). Prescribed exercise doses and intensities will be ramped over the initial 3 weeks of the study to allow for a gradual familiarization to the study protocol and the accompanying physical demands (see Table 1). Exercise intensity will be based on a percentage of the maximum heart rate (HR) achieved during the baseline maximal exercise test. The 12 kilocalories*kilogram of bodyweight^-1 ^* week^-1 ^(KKW) weekly exercise dose was chosen to approximate public health dose guidelines http://www.health.gov/paguidelines (i.e., approximately 30-50 min, 3-5 days per week). Participants will be scheduled to complete their weekly exercise dose over three exercise sessions. However, additional exercise sessions can be scheduled for those needing more than three sessions per week to achieve their target dose.

**Table 1:** Ramping Schedule for Exercise Dose and Intensity

**Week No**.	Exercise Dose (KKW)	Exercise Intensity **(% HR**_**max**_**)**
1	6	50 - 60
2	9	60 - 70
3 - 36	12	70 - 85

#### Exercise Sessions

During the supervised exercise sessions, participants will perform an active warm-up for 5 minutes prior to initiation of the exercise prescription. Polar RS 400 HR monitors (Kempele, Finland) will be used to continuously monitor participants' heart rate (HR) throughout the exercise session to ensure that they are within their target HR zone (i.e., 70-85% of an individual's HR_max_). Typically, participants can achieve a HR of 70-85% by either walking briskly at an incline or by running at zero incline. The speed and/or grade of the treadmill will be continually adjusted to maintain participant HR within the target HR zone while allowing the participants some flexibility in customizing their exercise session. A global Rating of Perceived Exertion (RPE), assessed using Borg's RPE scale, will be taken at the conclusion of each exercise session. At the completion of the exercise session, participants will perform an active cool down (0 grade and 50-70% of speed) until their HR returns to within 15% of their resting value. Participants will then be led through a 5-10 minute stretching routine.

During the continuation phase, participants will complete one supervised exercise session per week at the CTP exercise facility with the remainder of their exercise dose to be completed in unsupervised exercise sessions. Exercise Facilitators will aid the participant in identifying a safe location for completing their unsupervised sessions, scheduling these sessions, and planning other practical concerns related to engaging in exercise during the continuation phase. Participants will also receive assistance in developing measured exercise courses to allow for estimates of time and distance covered at each unsupervised session. Participants will record a global RPE score at the end of each unsupervised exercise session.

#### Exercise Safety

Those who are not safe to exercise, including those with greater than Stage 1 hypertension will not be randomized. Prior to each exercise session, participants will sit undisturbed for 5 minutes before assessment of their resting heart rate and blood pressure (BP). If a participant's resting HR is ≥ 100, it will be re-measured after an additional 5-minute rest period. If a participant's resting HR remains ≥ 100, the exercise session will be rescheduled for another day. Likewise, if a participant's resting BP is ≥ 160/100, it will be re-measured after an additional 5-minute rest period. If a participant's resting BP remains ≥ 160/100, the exercise session will be rescheduled for another day. During the session, BP will be assessed after 5 minutes to monitor for adverse responses to the onset of the exercise. BP will continue to be monitored if an exaggerated acute response is observed (i.e., SBP > 200 mmHg). The exercise session will be halted if an extreme BP response (SBP > 250 mmHg or remains unchanged despite an increase in workload, or DBP increase > 15 mmHg) is observed. Following the cool down, a final BP will be taken before the participant is cleared to leave the facility. Guidelines are also present for referral to appropriate medical care and additional physician clearance based on blood pressure readings.

### Health Education Intervention

The health education intervention is structured to provide equivalent contact time to that of the exercise intervention. During the acute phase, participants will complete three supervised sessions per week. Visits will be weekly during the continuation phase. During HEI sessions participants will view educational items such as didactic presentations, readings, Web sites, and audio or video materials. Instructional topics include areas such as healthy eating, preventive health care and recommended health screenings, accessing health care resources, and other health-related topics that are relevant to adults with substance use disorders. Exercise and topics central to substance use treatment (e.g., coping with cravings, relapse prevention) are not included to prevent intervention contamination and not change TAU, respectively. Participants will be encouraged to suggest topics of interest to help maintain their involvement and engagement in the sessions throughout the duration of the study. The HEI program is modeled after similar programs that have been used successfully as control groups in clinical trials examining exercise as an intervention [7,52,53].

### Behavioral Adherence Program

A comprehensive behavioral adherence approach to facilitating and monitoring fidelity to exercise dose has been developed by our team through our work in conducting exercise interventions in depressed populations [54]. Individuals in both treatment arms will receive adherence support through the behavioral adherence program. This program serves to: 1) provide necessary tools and support for participants to help them successfully complete the intervention and 2) allow for individualized monitoring to address person-specific adherence issues and barriers to the assigned intervention. The program includes: 1) multidisciplinary psychoeducation about adherence, 2) intervention skills training, 3) weekly intervention prescription provided via the study Web site, 4) self-monitoring of intervention adherence and performance, 5) adherence feedback from study Web site and intervention facilitator, and 6) weekly intervention planning (individually-tailored plan). Facilitators assist participants each week throughout the study with planning and adhering to a schedule for completion of their intervention sessions. Facilitators proactively review both potential and identified barriers to adherence (e.g., transportation, work schedules) and discuss possible solutions to work around these barriers (e.g., planning bus routes, providing flexible appointment times). Participants work with facilitators to use the study Web site during supervised sessions and are able to continue using the Web site during home-based sessions if they have access to the Internet.

### Study Assessments

Table 2 summarizes the assessments used throughout the study, their main purpose, their frequency of administration, and psychometric properties if available.

**Table 2:** STRIDE Assessments

Assessment	Frequency	Purpose and Psychometrics
*Diagnostic and Screening Measures*		

Demographics Form	Screen	To gather basic demographic information.

Composite International Diagnostic Interview (CIDI) (Modules A, J, & L)	Screen	To obtain substance use diagnoses. Tests of the reliability of the CIDI-SAM based on DSM-IV diagnoses for cocaine dependence compared to SCID interviews done by trained clinicians, had percent agreement of 82.6%, with kappa = 0.61. With specific criteria for the diagnosis, kappas ranged between 0.68 and 0.55.

Mini International Neuropsychiatric Interview (MINI)	Screen	To identify Axis I psychiatric diagnoses (excluding substance use disorders). In comparison to the Structured Clinical Interview for DSM-IV Disorders (SCID-P), kappa values were good (only one diagnosis < .50), specificities and negative predictive values were .85 or higher across diagnoses, and in general, sensitivity was .70 or higher [113].

Locator Form	Screen, monthly	To obtain contact information for each participant.

Prior and Concomitant Medications	Screen, weekly	To assess prescribed medications taken by the participant.

Self-Administered Comorbidity Questionnaire (SCQ)	Screen	To assess the presence of medical problems, their severity, and whether or not the condition limits functioning. An intraclass correlation coefficient of 0.94 shows good test-retest reliability and is comparable to the Charlson Index intraclass correlation coefficient of 0.92.

Physical Activity Readiness Questionnaire-Revised (PAR-Q)	Screen	To determine whether a person needs to consult with their physician prior to engaging in an exercise program.

Medical History- Self-report Form	Screen	To obtain information that will facilitate the conduct of the physical exam, clinician-rated medical history, and maximal exercise test.

Maximal Exercise Test Screening Questions	Screen	To aid the medical personnel in ensuring that it is safe for the participant to undergo the maximal exercise test

Maximal Exercise Testing	Screen, week 13	To examine cardiorespiratory responses in order to rule out ischemic response to exercise, to identify participants for whom exercise might be hazardous, and to provide data for the exercise prescription.

Physical Exam/Medical History	Screen	To provide clearance for exercise.

Laboratory Tests	Screen	To provide clearance for exercise.

*Substance Use and Treatment Assessments*		

Timeline Followback (TLFB)* *Primary outcome measure	Screen, 3X/wk for 1^st ^3 months, 1X/wk for next 6 months	To quantify days of substance use for calculation of primary outcome (percent days abstinent). The TLFB has been shown to have high test-retest reliability (ICC values ranging from 0.70 to .94, with all p < 0.001), good convergent and discriminate validity, and acceptable agreement between the TLFB and urine drug screens (Yule's Y of 87 or greater for amphetamines and cocaine)[114].

Urine Drug Screen (UDS)	Baseline, 3X/wk for 1^st ^3 months, 1X/wk for next 6 months	To test for substance use and to inform TLFB.

Stimulant Craving Questionnaire-Brief (STCQ-Brief)	Baseline, weekly	To assess current craving for stimulants. The CCQ-Brief, from which the STCQ-Brief is adapted, has high internal consistency, with Cronbach's alpha ranging from 0.87 [115] to 0.90 [61]. The instrument also has good construct validity and has shown to correlate well with other craving measures [115].

Stimulant Selective Severity Assessment (SSSA)	Baseline, weekly	To assess signs and symptoms of stimulant abstinence. The Cocaine Selective Severity Assessment, from which the SSSA is adapted, has been shown to have good inter-rater reliability (correlation coefficient = 0.92, p < 0.001) and internal consistency (Cronbach's alpha = 0.80).

Addiction Severity Index-Lite (ASI-Lite)	Baseline, weeks 13, 25, 37	To assess common problems associated with drug use. The CTN version is similar to the ASI-Lite-Veterans Administration (ASI-L-VA) and should have similar psychometric characteristics. Specifically, intraclass correlations between the ASI fifth edition (ASI-5) and ASSI-L-VA are 0.79 for alcohol, 0.79 for drug, 0.85 for legal, 0.46 for family/social, and 0.53 for psychiatric [116].

Fagerstrom Test for Nicotine Dependence (FTND)	Baseline	To assess dependence on nicotine. The FTND has shown acceptable internal consistency (Cronbach's alpha of 0.61) and correlates significantly with other measures of smoking consumption.

Treatment as Usual (TAU) Tracking Form	Baseline, weekly	To assess the participant's treatment for substance abuse within the past week.

*Measures of Mood, Sleep and Anhedonia*		

Quick Inventory of Depressive Symptomatology- Clinician rated version (QIDS-C_16_)	Baseline, weekly	To assess severity of depression-specific symptoms. The internal consistency coefficient is high (Cronbach's alpha of 0.90)[70]. It also has good concurrent validity, with correlations between the QIDS and the 17-item Hamilton Rating Scale for Depression ranging between .86 and .93. It also has been shown to have good inter-rater reliability with a kappa of .85.

Concise Health Risk Tracking- Self-report (CHRT-SR)	Baseline, weekly	To assess suicidality and related thoughts and behaviors. The CHRT-SR has good internal consistency (Cronbach's alpha of 0.78).

Concise Associated Symptoms Tracking- Self-report (CAST-SR)	Baseline, weekly	To assess symptoms related to suicidal thoughts and behaviors. The internal consistency coefficient for the CAST-SR is good (Cronbach's alpha of 0.77).

Snaith-Hamilton Pleasure Scale (SHAPS)	Baseline, monthly	To measure anhedonia, the inability to experience pleasure. The SHAPS has adequate construct validity, satisfactory test-retest reliability [117], and high internal consistency (Cronbach's alpha of 0.94)[117].

*Psychosocial Assessments*		

Short-Form Health Survey (SF-36)	Baseline, monthly	To assess quality of life and general health. Internal consistency reliability coefficients for the SF-36 are high (all greater than 0.80). Test-retest coefficients range from 0.43 to 0.90 for a 6-month interval and from 0.60 to 0.81 for a 2-week interval. The SF-36 has been shown to correlate moderately well with other health measures.

Quality of Life Enjoyment and Satisfaction Questionnaire Short Form (Q-LES-Q-SF)	Baseline, monthly	To evaluate general life enjoyment and satisfaction. Test-retest reliability for the Q-LES-Q-SF has been shown to be .86 [118] and internal consistency (Cronbach's alpha) has been shown to range from .86 to .90 [118,119].

Pain Frequency, Intensity and Burden Scale (P-FIBS)	Baseline, monthly	To evaluate the frequency, intensity, and burden of pain over the past week, as well as usage of pain medication to manage pain.

*Cognitive Function Assessments*		

Wechsler Test of Adult Reading (WTAR)	Baseline	To assess pre-morbid intelligence. The WTAR has been established to be a reliable and valid assessment of pre-morbid intelligence. It has been normed with the Wechsler Adult Intelligence Scale (WAIS-III) and the Wechsler Memory Scale (WMS-III).

MGH Cognitive and Physical Functioning Questionnaire (CPFQ)	Baseline, monthly	To assess physical well-being and cognitive and executive dysfunction. The CPFQ has been shown to have high internal consistency with a Cronbach's alpha of 0.90 and test-retest reliability (0.83, p < 0.001)[120].

Stroop Color and Word Test (Stroop)	Baseline, weeks 13, 37	To measure attention response inhibition.

*Physiological Measures*		

Physiological Measures	Baseline, monthly (height once at baseline, weight weekly)	To measure height, weight, body mass index (BMI), and waist circumference

Exercise Readiness Form	Baseline, each supervised exercise session (3X/wk for 1^st ^3 months, 1X/wk for next 6 months)	To measure resting heart rate and blood pressure for those in the exercise condition in order to evaluate safety for exercise.

*Retention*		

Treatment Participation Questionnaire (TPQ)	Baseline, weekly	To assess participant's likelihood of remaining in treatment.

#### Diagnostic and Screening Assessments

At screening, standard demographic information will be collected from all participants (e.g., gender, race, ethnicity, etc.). Contact information will be obtained and updated monthly and additionally as needed. DSM-IV-TR alcohol and substance abuse and dependence will be assessed with the substance use modules of the World Health Organization (WHO) Composite International Diagnostic Interview (CIDI) (Version 2.1)[55,56]. Additional DSM-IV Axis I diagnostic information will be obtained using the MINI International Neuropsychiatric Interview (MINI)[57].

#### Medical Evaluation

Medical history will be obtained using a self-report medical history form designed to collect information about history and/or treatment of medical conditions, such as heart disease, high blood pressure, diabetes, and cancer. The form also assesses allergies, past surgeries, tobacco and alcohol use, family history of medical conditions, medical symptoms over the past 30 days, and prior and concomitant medications. Additionally, participants will complete the Self-Administered Comorbidity Questionnaire (SCQ)[58] and the Physical Activity Readiness Questionnaire (PAR-Q)[59]. Medical personnel will conduct a medical history and physical exam, and laboratory tests (chemistry, hematology, lipid profile, and urinalysis) will be obtained as part of the medical screening process.

Maximal exercise testing will be conducted during the screening process to examine cardiorespiratory responses in order to rule out ischemic response to exercise (with its implications of cardiovascular disease), to identify participants for whom exercise might be hazardous, and to provide data for the exercise prescription. The test will be repeated at the end of the 12-week acute phase (or during an early termination visit). A trained technician will process the test data and a report will be generated that contains the following information: 1) participant's symptoms before, during, and after testing; 2) maximal heart rate achieved and percent of predicted maximal heart rate achieved; 3) time on treadmill and estimated maximal metabolic equivalent (METS) achieved; and 4) ECG interpretation. A designated medical staff member will be responsible for reviewing the participant's medical history and evaluating results from the medical exam, maximal testing, and lab results to determine whether or not the participant is medically cleared to exercise.

Eligible participants complete a comprehensive baseline assessment. Since there are numerous baseline assessments, these may be distributed over more than one day if necessary. The frequency and type of ongoing assessments are equivalent in both groups, with the exception of blood pressure and heart rate obtained for exercise participants prior to every supervised exercise session. Descriptions of ongoing study assessments are described by category below (as well as in Table 2).

#### Substance Use and Treatment Assessments

The Timeline Followback [48,49] and Substance Use Diary (SUD) will be used to acquire information on alcohol and drug use, including cocaine and other stimulants. The TLFB is a semi-structured interview that uses a calendar to prompt participants to provide retrospective estimates of their daily drug use over a specified period of time that can vary up to 12 months before the interview date. The measure provides prompts to facilitate accurate recollection of use behavior. The SUD, a pocket calendar given to participants at the beginning of the study to prospectively record substance use, will assist with accurate recall when completing the TLFB at each study visit.

Qualitative urine drug screens (UDS) will be conducted at baseline, three times a week in the first 12 weeks, and once a week in the subsequent 6 months. The screen will test for the following substances: marijuana, cocaine, opiates, amphetamine, methamphetamine, benzodiazepines, barbiturates, methadone, methylenedioxymethamphetamine (MDMA, Ecstasy), and oxycodone. UDSs will also be used to corroborate information on the TLFB.

The Addiction Severity Index-Lite (ASI-Lite) will be administered by research staff to examine multiple domains that are commonly affected by substance use, including medical, employment/self-support, alcohol/drug use, legal status, family/social, and psychiatric status [60]. Stimulant craving (cocaine, methamphetamine, and other stimulants) will be assessed using the Stimulant Craving Questionnaire-Brief (STCQ-Brief; adapted from Sussner et al. [61] and derived from the 10-item Cocaine Craving Questionnaire-Brief and the original 46-item Cocaine Craving Questionnaire-Now [62]). Signs and symptoms of stimulant (cocaine, methamphetamine, and other stimulants) abstinence will be evaluated using the Stimulant Selective Severity Assessment (SSSA; adapted from Kampman et al. [63]). Baseline nicotine dependence will be evaluated using the Fagerstrom Test for Nicotine Dependence (FTND)[64,65] and number of cigarettes smoked per day will be captured on the TLFB.

#### Mood and Functional Measures

Symptoms of depression will be measured by the Quick Inventory of Depressive Symptomatology - Clinician Rated version (16-item)[66-70], and the Snaith-Hamilton Pleasure Scale (SHAPS)[71] will be used to measure anhedonia. Suicidality and related symptoms will be evaluated throughout the study using the Concise Health Risk Tracking - Self-Report (CHRT-SR), and the Concise Associated Symptoms Tracking - Self-Report (CAST-SR).

Psychosocial assessments will include the Quality of Life Enjoyment and Satisfaction Questionnaire Short Form (Q-LES-Q-SF [72]), the Short-Form Health Survey (SF-36 [73,74]), and the Pain Frequency, Intensity and Burden Scale (P-FIBS). Assessments of cognitive function will include the Stroop Color and Word Test [75,76], a measure of attention response inhibition, and the MGH Cognitive and Physical Functioning Questionnaire (CPFQ [77]), a measure of subjective cognitive function. The Wechsler Test of Adult Reading (WTAR [78]) will be administered at baseline to assess premorbid intelligence.

#### Fitness and Physiological Measures

Physiological measures include height, weight, body mass index (BMI), and waist circumference. For those participants in the exercise intervention, resting heart rate and blood pressure will be obtained prior to each exercise session using the Exercise Readiness Form in order to evaluate safety to exercise. Resting heart rate and blood pressure will be obtained for all participants during the maximal exercise test. The maximal exercise will be repeated at week 13 to assess change in fitness.

#### Retention

The Treatment Participation Questionnaire (TPQ), a five-item self-report that assesses the participant's likelihood of remaining in treatment for substance abuse and continuing to attend study visits, will be administered at baseline and then weekly thereafter. Since this study is designed as an intent-to-treat study, subjects will be encouraged to complete assessments regardless of their status in their treatment programs or their engagement in the study intervention.

#### Analytic Methods

The primary analysis will compare the primary outcome between the two treatments taking into account possible variability in the overall level of abstinence between sites. More specifically, we use a mixed normal model with a fixed treatment effect and random terms for site and error. The model considers treatment effect to be the same in each site. P-value less than 0.05 will be considered statistically significant. The assumed treatment effect *μ_VIHD_*-*μ_HEC _*is 0.15. The error standard deviation *σ *is considered to be in the range between 0.40 and 0.45, and abstinence status within participant is assumed to be correlated across days. The primary endpoint will be analyzed on an intent-to-treat basis, meaning that participants' data will be analyzed according to the group they were randomized to, regardless of the subsequent sequence of events (e.g., noncompliance to the intervention).

Even though the overall level of outcome may differ across sites (i.e. *σ_site _*> 0), the primary analysis removes this variation by adjusting for site in the model. Hence, initial sample size computations are based on value of error standard deviation *σ *and the sample size estimates are based on a two sample t-test formula. A treatment effect that varies across sites may contribute additional variation and simulations with primary analysis performed for each simulation were used to investigate this possibility. Based on these simulations, and as an additional precaution, we increased initial sample size by 10% to attempt to take into consideration potential treatment effect variability. Based on these considerations, we proposed the trial to enroll 330 participants. Clearly, the proposed sample size depends on accurate estimation of variability *σ*. We propose to reassess this variability midway through the study, i.e., after approximately 165 participants have enrolled and completed the acute phase of the trial, and may readjust needed sample size if the variance assumption proves incorrect.

## Rationale for Key Study Design Choices

### Population

For this study, we chose to specifically evaluate individuals with stimulant abuse or dependence. This decision was made in order to reduce heterogeneity of the sample with respect to drug use, since this is an early efficacy trial with a new intervention in a substance using population.

### Setting

This study is designed to start while participants are in a residential setting and continues as participants transition into outpatient and other community care. Starting treatment in the residential setting was chosen to maximize the availability of participants to be present for the initial evaluation and training of the exercise intervention. Given the dose and intensity of exercise selected for this study, it is critical that participants be consistent in attending supervised sessions, particularly as they are first learning how to implement the exercise protocol, how to use all related equipment, and how to collect and record all exercise-related data. A transition to outpatient care is a typical occurrence in the treatment of substance use disorders. This study is designed to evaluate participants as they transition to the next phase of care. While the decision to begin in a residential setting could result in somewhat less generalizability of findings, since this is an early efficacy study, we determined that it was more important to ensure that our study population was present to receive sufficient training and supervision in the exercise intervention and to maximize adherence during this time.

### Study Duration

The total duration of participation in STRIDE is 9 months. A 6-month duration for the continuation phase was selected because we would like to see if there is a sustained response to the exercise intervention. In most chronic diseases, longer-term outcomes are best seen in the 6 months following acute phase intervention.

### Exercise Intervention

One of the major hurdles in successfully conducting exercise trials has been the inconsistency of type, dose, and intensity of exercise achieved. We elected to define exercise type, dose and intensity in this study to minimize variability and to achieve pre-specified targets for both dose and intensity.

#### Type

We selected the use of aerobic exercise in this trial. While there is some evidence that other types of exercise (e.g., weight training) may be beneficial to individuals with other disorders, such as depression, the preponderance of existing evidence supports the use of aerobic exercise. Furthermore, aerobic exercise has been used in previous interventions involving substance abuse disorders [7-11,18].

#### Dose

A 12 KKW dose of exercise was selected for this study for the following reasons: 1) it is consistent with U.S. 2008 Physical Activity Guidelines http://www.health.gov/paguidelines, as well as prior recommendations from groups such as the Centers for Disease Control and Prevention and ACSM [79-83], the NIH Consensus panel [45,79,80,82-86], and the U.S. Surgeon General (U.S. Department of Health and Human Services [87]); 2) it has been used in previous studies involving physical activity interventions in smokers [7,9], and in other disorders such as depression [54,88-91], it is consistent with doses associated with biological changes that we hypothesize will impact stimulant use such as alterations of the serotonergic [92-94] and endocannabinoid systems [95], attenuated stress responses [96,97], and increases in release of BDNF [98].

#### Intensity

An intensity range of 70-85% of an individual's HR_max _was selected for this study. This intensity is above the suggested minimum intensity of 50% of HR_max _and within the currently recommended training intensity guidelines for producing physiological adaptations and benefits [51]. Although higher exercise intensities may produce greater changes in outcomes at a constant dose, for this efficacy trial we want to be certain that we are well within standard exercising ranges for both dose and intensity. By selecting a range of 70-85% of HR_max_, we have allowed for some exercising flexibility to increase comfort, while ensuring that all participants are exercising above the minimum recommended threshold.

Alternatively, we could have allowed participants to select their own exercise intensity (i.e., self-selected intensity based on rating of perceived exertion) as long as they achieved the target dose. While this approach would allow significantly greater flexibility for participants, it would likely lead to greater variations in exercise intensity. Another alternative would be to have a fixed exercise intensity (i.e., 80% HR_max_) to maximize the standardization of the exercise intervention. However, a fixed intensity would allow little flexibility for participants to find a comfortable exercise level and may, therefore, result in poor adherence and increased drop-outs.

#### Frequency

In this study, we recommend that participants complete their prescribed weekly exercise dose over three exercise sessions per week. However, we will allow up to two additional sessions per week in order to accommodate variations in baseline fitness levels. Individuals who are more unfit will have a relatively low caloric expenditure rate when exercising within their relative intensity range (as demonstrated by elevated HRs at low treadmill settings). This will require them to exercise for a greater duration to achieve their weekly exercise dose than individuals who have higher baseline fitness levels. For example, a participant exercising at 70% of their HR_max _might require 150 min/week (50 min/session for three sessions/week) to achieve the 12 KKW dose, whereas a less fit participant might require 240 min/week to achieve the same 12 KKW dose due to differences in exercise efficiency and their individual responses to exercise. Therefore, it benefits the second participant to divide their exercise dose among four or five sessions, thereby increasing the feasibility of completing each exercise session and achieving the weekly exercise dose.

### Control Group

A health education intervention attention control group was selected so that the effect of the exercise intervention could not be attributed to the additional contact associated with it. HEI has been established as an ineffective, yet attentionally equivalent control condition in studies of exercise and it has been used successfully [7,52,53,99,100] and with good adherence by members of our study Protocol Development Team (Drs. Marcus, Church, Nunes and Blair). Their use of HEI as a control condition and the knowledge gained from their experience increases the likelihood that participants randomized to this arm of the study will find this intervention acceptable. Participants will be recruited to a "health intervention study", one arm of which is exercise, and the other health education, so that HEI is not described as a control condition. While it is true that participants will know their group assignment, this has not been problematic in other studies using HEI controls and there has not been differential attrition (i.e., Marcus et al. [7,10]). Participants in the HEI group will be able to participate in selecting the material that is discussed in order to help maintain interest. Furthermore, they will be monitored similarly to DEI participants, and they will receive the same behavioral intervention strategies as DEI participants in order to maximize adherence. All attempts will be made to address barriers to retention in the control condition, as in the exercise intervention. HEI, as opposed to TAU alone, is also intended to ensure continued participant engagement in the study and thus minimize dropouts that might occur if attention in the two groups were not equivalent. Other possible active control conditions (e.g., resistance training, relaxation, yoga, or meditation) have the potential for efficacy, and in fact some have shown efficacy both in substance use and in other disorders (e.g., depression [101]). Thus, the use of an active comparator would make it difficult to see a treatment effect, so these alternative active control conditions are not ideal comparators for an efficacy study of exercise.

### Primary Outcome

The rationale for the outcome for the current study design was driven by the desire to provide a meaningful clinical outcome that would allow for the ready comparison of the effectiveness of this treatment. In addition, it was important that this outcome be suitable for an intent-to-treat study such that all available data could be utilized for analysis. We decided upon an approach that integrates the strengths of multiple measures and allows for data collection over an extended time period [46]. We chose to assess percent days abstinent as measured by self-report using the Timeline Followback. Some studies have shown that this approach has been shown to correlate well with objective measures of use such as urine drug screen [102-104]. Disadvantages of the TLFB include potential inaccuracy due to memory errors and bias and deliberate and denial-based distortions of reported substance use. As a result, two additional tools have been added to the data collection process. The Substance Use Diary, which will aid with recall during the TLFB assessment, has been shown to improve the accuracy of the TLFB [105]. The diary can be completed in real time in between study visits to help participants maintain an accurate accounting of their usage of stimulants, and will be particularly helpful in situations where missed visits occur that result in greater amounts of time to recall. Finally, to further improve the validity of the TLFB, results will be confirmed with urine drug screens collected 3 times per week and an algorithm will be employed at the time of data analysis to reconcile discrepancies between the TLFB and the urine drug screens for the primary outcome.

Choice of the TLFB self-report measure was consistent with several elements of our study design. Our study intervention will be conducted over a 9-month period during which time participants will not only transition from residential to outpatient treatment, but they are expected to come to the study site 3 times a week for the first 12 weeks and once a week for the following 24 weeks. Stimulant abusing patients are known to be inconsistent in their attendance at treatment and study visits [1]. This visit schedule therefore poses a serious challenge to the use of a sole objective measure such as UDS to define abstinent and non-abstinent days and would likely result in considerable missing data. To decrease the potential for missing data due to the short half-life of stimulants, urine drug screens are often obtained 3 times per week, but this frequency of assessment would be very difficult to maintain over a 9-month duration. The TLFB, on the other hand, allows for data to be collected on all days, since the interviewer prompts the participant to recall use for all days since the last study visit.

## Discussion

The STRIDE study aims to address the need for novel treatments in stimulant use disorders. Studies demonstrating the effectiveness of exercise in the treatment of nicotine dependence and alcohol abuse suggest that exercise augmentation of treatment as usual may be efficacious in increasing abstinence from stimulant use. The potential effects of exercise on the underlying biological mechanisms of stimulant addiction further support the use of exercise in the treatment of stimulant abuse. Finally, exercise may provide additional health benefits. Stimulant use is detrimental to a number of health domains including sleep [106,107], cognitive function [36], and mood [108], while exercise results in improvement of sleep quality [26,109], cognitive function [110], and mood [89,94,111,112].

Several study design features help accurately assess the efficacy and effectiveness of exercise augmentation for stimulant abuse and dependence. First, the exercise type, dose, and intensity are precisely defined to reduce variability. In addition, this dose of exercise is congruent with current health recommendations and should be sufficient to alter the hypothesized biological underpinnings of stimulant dependence. Second, a comprehensive behavioral intervention program will be implemented to facilitate and monitor adherence to the two interventions. Facilitators will provide participants with adherence feedback and support the participant in addressing barriers to adherence. Finally, the use of a health education intervention control group will provide balanced facilitator attention across the two interventions. Health education groups have proved to be ineffective in previous trials [7,52,53,99], yet demonstrate high levels of adherence and will provide equivalent facilitator contact.

This is one of the first studies conducted within the CTN that is specifically designed as a first test of a new intervention in a specific disorder. As such, it will not only test the efficacy and effectiveness of this intervention for stimulant abuse and dependence, but also provide information about conducting this type of study in the context of a network of community treatment programs. Future studies are needed that evaluate the use of exercise in the reduction in use of other illicit substances. In addition, new studies that examine the use of exercise during different phases of treatment are needed. For example, exercise may prove to be effective in reducing symptoms of withdrawal but not in increasing long-term abstinence, or vice versa. Finally, future studies should aim to identify the biological mechanisms responsible for reductions in substance use.

## Competing interests

Madhukar H. Trivedi, M.D. is a consultant to or on speaker bureaus for Abbott Laboratories, Inc., Abdi Ibrahim, Akzo (Organon Pharmaceuticals Inc.), AstraZeneca, Bristol-Myers Squibb Company, Cephalon, Inc., Cyberonics Inc., Eli Lilly & Company, Evotec, Fabre Kramer Pharmaceuticals, Inc., Forest Pharmaceuticals, GlaxoSmithKline, Janssen Pharmaceutica Products, LP, Johnson & Johnson PRD, Meade Johnson, Medtronic, Neuronetics, Otsuka Pharmaceuticals, Parke-Davis Pharmaceuticals, Inc., Pfizer Inc., Sepracor, SHIRE Development, Solvay Pharmaceuticals, VantagePoint, and Wyeth-Ayerst Laboratories. He receives research support from the Agency for Healthcare Research and Quality (AHRQ), Corcept Therapeutics, Inc., Cyberonics, Inc., Merck, National Alliance for Research in Schizophrenia and Depression, National Institute of Mental Health, National Institute on Drug Abuse, Novartis, Pharmacia & Upjohn, Predix Pharmaceuticals (Epix), Solvay Pharmaceuticals, Inc., and Targacept.

Tracy L. Greer Ph.D. has received research support from the National Alliance for Research in Schizophrenia and Depression.

Bruce D. Grannemann, M.A. declares that there is no conflict of interest.

Timothy S. Church, M.D., Ph.D., M.P.H. declares that there is no conflict of interest.

Eugene Somoza, M.D., Ph.D. declares that there is no conflict of interest.

Steven N. Blair, P.E.D. declares that there is no conflict of interest.

Jose Szapocznik, Ph.D. declares that there is no conflict of interest.

Mark Stoutenberg, Ph.D. declares that there is no conflict of interest.

Chad Rethorst, Ph.D. declares that there is no conflict of interest.

Diane Warden, Ph.D., M.B.A. has owned stock in Bristol Myers Squibb and Pfizer, Inc. in the last 5 years and has received funding from the National Alliance for Research in Schizophrenia and Depression.

David W. Morris, Ph.D. declares that there is no conflict of interest.

Andrzej S. Kosinski, Ph.D. declares that there is no conflict of interest.

Tiffany Kyle, Ph.D. declares that there is no conflict of interest.

Bess Marcus, Ph.D. declares that there is no conflict of interest.

Becca Crowell, M.Ed., Ed.S. declares that there is no conflict of interest.

Neal Oden, Ph.D. declares that there is no conflict of interest.

Edward Nunes, M.D. has received funding from NIDA for grants K24DA022412 (PI: Nunes) and U10DA13035 (PI: Nunes).

## Authors' contributions

MHT, TLG, and BDG designed the trial, with substantial input from ES, JS, EN, DW, TSC, SNB, TK, BHM, and BC. ASK and NO calculated power and developed plans for statistical analyses. MHT, TLG, BDG, DW, and KMR formalized the procedures for study implementation, with substantial contributions from RW, DWM, ES, and EN on the selection, administration, and training of study assessments. Participant eligibility and recruitment procedures were developed by BDG, TLG, DW, RW, DM, TK, and BC. BDG, DW, TLG, and KMR determined site selection criteria with substantial input from TK and BC. CR, MS, TSC, TLG, BHM, MHT, and SNB developed the exercise intervention. KMR, CR, MS, TSC, TLG, BHM, MHT, and SNB developed the health education intervention. BDG, TLG, MHT, CR, DW, SNB, and KMR developed the behavioral adherence program. All authors contributed to revisions of the manuscript prior to submission and read and approved the final manuscript.
